# Healthcare practitioners experiences in delivering sexual and reproductive health services to unmarried adolescent clients in Jordan: results from a cross-sectional survey

**DOI:** 10.1186/s12913-021-07415-y

**Published:** 2022-01-05

**Authors:** Neena R. Kapoor, Ana Langer, Areej Othman, Jewel Gausman

**Affiliations:** 1 Women & Health Initiative; Department of Global Health and Population, Harvard T. H. Chan School of Public Health, 655 Huntington Ave, Boston, MA USA; 2 Maternal and Child Health Nursing Department; School of Nursing, University of Jordan, Amman, Jordan

**Keywords:** Jordan, Adolescents, Sexual and reproductive health, Youth, Family planning, Health services

## Abstract

**Background:**

The need for youth-friendly sexual and reproductive health (SRH) services has been identified as a national policy priority in Jordan, but there remains limited data on service utilization among adolescents, especially those who are unmarried, and there is limited training for healthcare practitioners (HCPs) in providing SRH services to youth. The objectives of this study are to 1) describe the most common reasons for encounters that HCPs have with unmarried youth clients about SRH topics and 2) explore differences in SRH services provided to unmarried youth by provider in Jordan.

**Methods:**

This cross-sectional study used a two-stage cluster-randomized sampling scheme to sample HCPs (doctors, nurses, and midwives) from health facilities in four governorates in Jordan. Data were collected on practitioner demographics, facility characteristics, and self-reports of having provided services related to nine common SRH concerns to unmarried girls or boys between the ages of 15–19 years. Chi-square tests were conducted to analyze the associations between provider and facility characteristics, client sex, and types of services rendered.

**Results:**

In total, 578 providers participated in the study (110 male and 468 female). Practitioners most commonly reported seeing unmarried female youth for concerns related to puberty (38.5%) and family planning (18.51%) and unmarried male youth for concerns of puberty (22.49%) or condoms (11.59%). In total, 64.45, 64.61 and 71.19% of midwives, nurses, and doctors reported having provided any SRH service to an unmarried adolescent. While practitioners most often reported seeing clients of the same sex, male practitioners were more likely to report having seen a female client for STIs (9.09% vs. 4.27% *p* = 0.040), and providing general information about sexual activity (12.73% vs. 5.77% *p* = 0.011) than female providers.

**Conclusions:**

Our results suggest that a substantial proportion of HCPs have provided SRH services to unmarried youth – challenging existing perceptions of the SRH care-seeking practices of unmarried youth in this conservative context.

## Introduction

Accessing high quality sexual and reproductive health (SRH) services is a common challenge faced by youth around the world. In many low-resource settings, health systems and policy limitations combined with cultural norms often prevent youth from receiving appropriate and timely services [1]. The need for youth friendly SRH services has been identified as a national priority in Jordan, but the country lacks both the necessary health services and policy infrastructure, as well as an enabling cultural environment, to establish services specifically targeting the needs of youth [2]. In the past 10 years, Jordan has undergone both demographic and cultural changes due to its growing population of youth, increasing average age of marriage, and rising refugee population, which necessitates establishing more robust SRH services for youth [3].

The Arab region has been recognized as falling short of meeting the SRH needs of youth, especially unmarried youth, partially due to the cultural context [4, 5]. Previous research suggests that youth in Jordan feel ashamed when seeking SRH services or information due to the conservative social environment [6]. Furthermore, youth in Jordan are dissatisfied with the quality of SRH service delivery, expressing concerns about both the quality of care and the poor interpersonal communication skills of healthcare practitioners (HCPs), resulting in underutilization of these services [7]. While new youth-friendly service delivery guidelines are currently under development, providers may not be aware of existing service delivery guidelines for youth clients [2]. Given these limitations, the demand for SRH information and services among youth is not currently being met.

In Jordan, the conservative context prohibits sexual activity among unmarried youth due to the high social and religious values placed on virginity [7]. Due to cultural taboos, very little research exists on the SRH needs of unmarried youth and interventions to address them. Unmarried adolescents are, however, still at risk of morbidity and mortality from a lack of appropriate and timely SRH services [2]. A study conducted in 1994 in Jordan found that 7% of university students reported premarital sexual activity, though these data are both limited and outdated [4]. The restricted research on SRH issues conducted among unmarried youth in Jordan suggests that youth have little knowledge of reproductive health and fear shame and punishment if they were to seek SRH information or services [6]. A qualitative study in Jordan found that parents are ashamed to discuss SRH issues with their children and lack confidence in their own knowledge [8]. Further, SRH topics are rarely addressed in the educational context in Jordan as there is no standard curriculum and teachers are often unprepared to discuss such topics [2, 4, 9]. Thus, HCPs in this context need to play an important and essential role in ensuring that youth have access to knowledgeable practitioners who are equipped to meet their needs.

The overall objective of this study was to better understand HCPs’ experiences in delivering services to unmarried adolescent clients while specifically 1) describing the most common reasons for encounters that HCPs have with this segment of the population about SRH topics and 2) exploring differences in SRH services provided to female and male unmarried youth by provider in Jordan. These results provide important insight that could help improve SRH service delivery to this especially vulnerable, and understudied, population. In addition, these results could offer insight on improvements to policy environment in health facilities and training for providers. Furthermore, given both the logistical and cultural constraints in conducting research on SRH among unmarried youth directly in Jordan, this research offers an alternative perspective on their SRH needs.

## Methods

The data were obtained in a cross-sectional study conducted in primary health, comprehensive care, and maternal and child health centers in four governorates in Jordan: Amman, Irbid, Mafraq, and Zarqa. We used a two-stage cluster sampling scheme. In the first stage, health facilities were randomly selected from all public health facilities in each of the four governorates. In the second stage, study participants were recruited by convenience from primary care physicians, midwives and nurses working in those facilities. All providers were eligible to participate. The final sample was determined using the following equation to calculate the per-governorate sample size:

$$N=\left(\left({t}^2\ast p\left(1-p\right)\right)/m\hat{\mkern6mu} 2\right)\ast {D}_{eff}$$

where: N = required sample

t = value of confidence level

p = estimated prevalence of the indicator of interest

m = margin of error

D_eff_ = intra-cluster correlation

Based on the above, using a 95% CI (so that t = 1.96) and a margin of error of 0.1, assuming *p* = 0.5 to maximize the sample requirements given that the real value of p is unknown, and an intra-cluster correlation of 1.2, as is common in facility-based surveys, the sample size needed was determined to be 116 HCPs per governorate, Thus, our study required a total sample size of 464 HCPs.

The demographic portion of the survey contained questions on practitioner age, sex, marital status, type of facility, religion, governorate, rural/urban residence, position, years of experience, training on reproductive health and awareness of guidelines that pertain to the provision of reproductive health services. The survey questions regarding SRH concerns asked if the HCP had ever encountered an unmarried adolescent girl or boy between the ages of 15 or 19 who asked about concerns related to: sexually transmitted infections or HIV, puberty, biological concerns, family planning/contraception, condoms, general information about sexual activity, romantic relationships between partners, and parent-child communication about sexual and reproductive health issues. Providers could select multiple concerns. The survey was pre-tested, validated and finalized in a separate pilot study with 50 HCPs in facilities in Amman.

Data collectors participated in trainings on research ethics and study procedures prior to the start of the study. Survey data was entered in real-time by study staff and reviewed by a study investigator to ensure that data obtained was of high quality and entered accurately. Ethical approval was obtained from the Institutional Review Boards at the Harvard T.H. Chan School of Public Health and the University of Jordan School of Nursing. Ethical approval and permission to conduct the research was also obtained from the Jordanian Ministry of Health. Informed consent was obtained from all participants before administering the survey.

Descriptive analyses were conducted to explore practitioner demographics and the most common concerns that HCPs reported amongst youth clients. A variable was constructed to indicate whether a HCP saw an unmarried youth for any of the SRH concerns specified. Chi-square tests were conducted to assess the association between HCPs reporting having encountered either male or female youth for SRH concerns and the provider or facility characteristics.

## Results

Table 1 presents provider characteristics. 578 HCPs participated in the study, of which 110 were male and 468 were female. The participants were nurses (42.41%), midwives (36.82%) and primary care physicians (20.59%). The HCPs primarily worked either at a primary/comprehensive care center (56.75%) or maternal and child health center (41.35%). Male practitioners were most commonly primary care physicians (75.45%) while female practitioners were midwives (45.36%) or nurses (47.08%). At the time of the survey, the HCPs were most likely to be 25 to 45 years of age (77.47%) and have less than 10 years of work experience (55.59%). The majority of HCPs also reported that they had training on reproductive health (62.06%) and were aware of guidelines on adolescent SRH (54.97%).

**Table 1:** Provider Demographic Characteristics

	Male HCPs	Female HCPs	Total
Number of Practitioners	110	468	578
Age			
18–24	0.91% (1)	10.06% (47)	8.32% (48)
25–35	50.91% (56)	48.82% (228)	49.22% (284)
36–45	19.09% (21)	30.41% (142)	28.25% (163)
46 or older	28.18% (31)	10.71% (50)	14.04% (81)
Prefer not to answer	0.91% (1)	0% (0)	0.17% (1)
Marital Status			
Married	80.91% (89)	79.06% (370)	79.41% (459)
Single	19.09% (21)	19.87% (93)	19.72% (114)
Prefer not to answer	0% (0)	1.07% (5)	0.87% (5)
Facility Type			
Maternal Child Health Center	20.91% (23)	46.15% (216)	41.35% (239)
Primary/Comprehensive Care center	75.45% (83)	52.35% (245)	56.75% (328)
Prefer not to answer	3.64% (4)	1.5% (7)	1.9% (11)
Religion			
Muslim	98.18% (108)	98.93% (463)	98.79% (571)
Christian	1.82% (2)	1.07% (5)	1.21% (7)
Governorate			
East Amman	40.91% (45)	38.89% (182)	39.27% (227)
Irbid	16.36% (18)	21.15% (99)	20.24% (117)
Mafraq	27.27% (30)	20.09% (94)	21.45% (124)
Zarqa	15.45% (17)	19.87% (93)	19.03% (110)
Location Type			
Rural	41.28% (45)	32.54% (150)	34.21% (195)
Urban	57.8% (63)	67.46% (311)	65.61% (374)
Other response	0.92% (1)	0% (0)	0.18% (1)
Practitioner Type			
Midwife	0.91% (1)	45.36% (210)	36.82% (211)
Nurse	22.73% (25)	47.08% (218)	42.41% (243)
Primary Physician	75.45% (83)	7.56% (35)	20.59% (118)
Other response	0.91% (1)	0% (0)	0.17% (1)
Years of Practice			
Less than 5 years	26.36% (29)	27.06% (125)	26.92% (154)
5–10 years	32.73% (36)	27.71% (128)	28.67% (164)
11–20 years	22.73% (25)	34.42% (159)	32.17% (184)
More than 20 years	18.18% (20)	10.82% (50)	12.24% (70)
Ever Received Training on SRH			
Yes	46.36% (51)	65.8% (304)	62.06% (355)
No	53.64% (59)	33.98% (157)	37.76% (216)
Other response	0% (0)	0.22% (1)	0.17% (1)
Aware of SRH Service Delivery Guidelines			
Yes	52.73% (58)	55.51% (257)	54.97% (315)
No	47.27% (52)	44.49% (206)	45.03% (258)

Table 2 reports the percentage of HCPs that reported encountering an unmarried male or female adolescent client for any of the following SRH concerns: puberty, family planning, biological concerns, parent child communication about SRH issues, sexually transmitted infections, condoms, general information about sexual activity, and romantic relationships between partners. 62.28% of HCPs saw a female client for any concern, while 37.89% of HCPs reported seeing a male client for any concern. Practitioners most frequently reported seeing female clients in visits related to puberty (38.58%) and family planning (18.51%). For male clients, HCPs most frequently reported seeing clients for concerns regarding puberty (22.49%) or sexually transmitted infections (8.13%).

**Table 2:** Percentage of practitioners who reported providing SRH services to unmarried male and female youth by topic

	Percent of HCPs having seen at least one female youth (n)	Percent of HCPs having seen at least one male youth (n)
Number of providers	578	578
Puberty	38.58% (223)	22.49% (130)
Family Planning	18.51% (107)	5.71% (33)
Biological Concerns	12.98% (75)	6.93% (40)
Parent child communication about SRH issues	13.15% (76)	6.06% (35)
Sexually transmitted infections	5.19% (30)	8.13% (47)
Condoms	5.02% (29)	6.57% (38)
General information about sexual activity	7.09% (41)	6.92% (40)
Romantic relationships between partners	3.98% (23)	3.98% (23)
Any concern	62.28% (360)	37.89% (219)

Chi-square tests were conducted to examine associations between provider demographics and client sex (Table 3). In total, 64.45, 64.61 and 71.19% of midwives, nurses, and physicians, respectively, reported seeing unmarried youth clients with regard to SRH concerns. Overall, a larger percentage of female practitioners reported having provided services to a female client for any SRH concern compared to male HCPs (64.53% vs. 52.73%, *p* = 0.022), while a greater percentage of male HCPs reported provided services to a male client for any SRH concern than female HCPs (63.64% vs. 31.84%, *p* < 0.001). A larger percentage of HCPs who had received training on SRH reported encountering a female client for SRH services than those who had not received training on SRH (65.63% vs. 56.48%, *p* = 0.04). Additionally, a greater percentage of HCPs who reported that they were aware of SRH guidelines reported having provided SRH services to both female and male clients than those who were not aware of such guidelines (73.65% vs. 48.06%, *p* < 0.001; 46.35% vs. 26.74%, *p* < 0.001). A larger percentage of younger HCPs, aged 18–24 years, reported seeing female clients compared to HCPs ages 25–35 years, 36–45 years or 46 years or older (81.25% vs. 60.21% vs. 58.28% vs. 66.67%, *p* = 0.024). A larger percentage of primary care physicians reported seeing male clients compared to midwifes or nurses (58.47% vs. 24.64% vs. 38.27%, *p* < 0.001). Similarly, a larger percentage of HCPs in primary comprehensive care centers reported providing care to male clients compared to HCPs working in maternal child health centers (45.12% vs. 27.62%; *p* < 0.001), while a larger percentage of HCPs in Zarqa reported seeing an unmarried adolescent client of either sex compared to other geographic areas.

**Table 3:** Percentage of practitioners reporting providing services to unmarried male and female youth according to provider characteristics

	Percent of HCPs having seen at least one female youth (*n* = 578)			Percent of HCPs having seen at least one male youth (*n* = 578)			Percent of HCPs having seen at least one youth (*n* = 578)		
Number of Practitioners	Yes % (N)	No % (N)	*p*-value	Yes % (N)	No % (N)	p-value	Yes % (N)	No % (N)	p-value
Age									
18–24	81.25%(39)	18.75% (9)	0.024	41.67% (20)	58.33%(28)	0.777	83.33%(40)	16.67% (8)	0.026
25–35	60.21%(171)	39.79%(113)		36.97%(105)	63.03%(179)		63.73%(181)	36.27%(103)	
36–45	58.28%(95)	41.72%(68)		36.20%(59)	63.8%(104)		62.58%(102)	37.42%(61)	
46 or older	66.67%(54)	33.33%(27)		41.98%(34)	58.02%(47)		71.6%(58)	28.4% (23)	
Gender									
Male	52.73%(58)	42.27%(52)	0.022	63.64%(70)	36.36%(40)	< 0.001	69.09%(76)	30.91%(34)	0.460
Female	64.53%(302)	35.47%(166)		31.84%(149)	68.16%(319)		65.38%(306)	34.62%(162)	
Marital Status									
Married	59.91%(275)	40.09%(184)	0.060	36.38%(167)	63.62%(292)	0.254	64.09%(296)	35.51%(163)	0.234
Single	71.93%(82)	28.07%(32)		42.98%(49)	57.02%(65)		72.81%(83)	27.19%(31)	
Facility Type									
Maternal Child Health Center	59%(141)	41%(98)	0.191	27.62%(66)	72.38%(173)	< 0.001	61.51%(147)	38.49%(92)	0.1
Primary/Comprehensive Care Center	64.02%(210)	35.98%(118)		45.12%(148)	54.88%(180)		68.9%(226)	31.1%(102)	
Religion									
Muslim	62.7%(358)	37.3%(213)	0.064	38.18%(218)	61.82%(353)	0.195	66.55%(380)	33.45%(191)	0.035
Christian	28.57% (2)	71.43% (5)		14.29% (1)	85.71% (6)		28.57% (2)	71.43% (5)	
Governorate									
East Amman	61.23%(139)	38.77%(88)	0.046	44.93%(102)	55.07%(125)	0.001	66.96%(152)	33.04%(75)	0.066
Irbid	57.26%(67)	42.76%(50)		35.9%(42)	64.1%(75)		61.54%(72)	38.46%(45)	
Mafraq	58.87%(73)	43.13%(51)		23.39%(29)	76.61%(95)		60.48%(75)	39.52%(49)	
Zarqa	73.64%(81)	26.36%(29)		41.82%(46)	58.81%(64)		75.45%(83)	24.55%(27)	
Location Type									
Rural	58.97%(115)	41.03%(80)	0.408	31.79%(62)	68.21%(133)	0.065	62.56%(122)	37.44%(73)	0.398
Urban	63.64%(238)	36.36%(136)		40.11%(150)	59.89%(224)		67.38%(252)	32.62%(122)	
Practitioner Type									
Midwife	63.51%(134)	36.49%(77)	0.335	24.64%(52)	75.36%(159)	< 0.001	64.45%(136)	35.55%(75)	0.288
Nurse	63.37%(154)	36.63%(89)		38.27%(93)	61.73%(150)		64.61%(157)	35.39%(86)	
Primary Physician	56.78%(67)	43.22%(51)		58.47%(69)	41.53%(49)		71.19%(84)	28.81%(34)	
Years of Practice									
Less than 5 years	69.48%(107)	30.52%(47)	0.045	44.81%(69)	55.19%(85)	0.174	74.03%(114)	25.97%(40)	0.021
5–10 years	56.1%(92)	43.9%(72)		34.76%(57)	65.24%(107)		59.15%(97)	40.85%(67)	
11–20 years	63.59%(117)	36.41%(67)		34.24%(63)	65.76%(121)		67.39%(124)	32.61%(60)	
More than 20 years	54.29%(38)	45.71%(32)		35.71%(25)	64.29%(45)		58.57%(41)	41.43%(29)	
Ever Received Training on SRH									
Yes	65.63%(233)	34.37%(122)	0.040	38.31%(136)	61.69%(219)	0.645	68.17%(242)	31.83%(113)	0.145
No	56.48%(122)	43.52%(94)		36.11%(78)	63.89%(138)		62.5%(135)	37.5%(81)	
Aware of SRH Service Delivery Guidelines									
Yes	73.65%(232)	26.35%(83)	< 0.001	46.35%(146)	53.65%(169)	< 0.001	76.83%(242)	23.17%(73)	< 0.001
No	48.06%(124)	51.94%(134)		26.74%(69)	73.26%(189		52.71%(136)	47.29% (122)	

Tables 4 and 5 further explore the association between provider-client sex in relation to different youth SRH concerns. A larger percentage of female HCPs reported seeing a female client for puberty-related concerns (40.60% vs. 30.00% *p* = 0.040), while a larger percentage of male HCPs reported seeing a female client for STIs (9.09% vs. 4.27% *p* = 0.040), and general information about sexual activity compared to female HCPs (12.73% vs. 5.77% *p* = 0.011). Approximately the same proportion of male and female HCPs reported seeing a female client for family planning services. A larger percentage of male HCPs reported seeing male clients for the following concerns as compared to female HCPs: puberty (36.36% vs. 19.23% *p* < 0.001), biological concerns (13.64% vs. 5.35% *p* = 0.002), STIs (26.36% vs. 3.85% *p* < 0.001), condoms (19.09% vs. 3.63% *p* < 0.001), general information about sexual activity (23.64% vs. 2.99% *p* < 0.001), romantic relationships between partners (10.91% vs. 2.35% *p* < 0.001).

**Table 4:** SRH concerns among unmarried female youth reported by provider, according to sex

Youth SRH Concern	Percent of male HCP reporting having seen a female client % (n)	Percent of female HCP reporting having seen a female client % (n)	p-value
Number of HCPs	110	468	
Puberty	30.00%	40.60%	0.040
Family Planning	20.91%	17.95%	0.472
Biological Concerns	16.36%	12.18%	0.240
Parent child communication about SRH issues	13.64%	13.03%	0.866
Sexually transmitted infections	9.09%	4.27%	0.040
Condoms	8.18%	4.27%	0.091
General information about sexual activity	12.73%	5.77%	0.011
Romantic relationships between partners	5.45%	3.63%	0.379
Any concern	52.73%	64.53%	0.022

**Table 5:** SRH concerns among unmarried male youth reported by provider, according to sex

Youth SRH Concern	Percent of Male HCPs reporting having seen a male client % (n)	Percent of Female HCPs reporting having seen a male client % (n)	p-value
Number of HCPs	110	468	
Puberty	36.36%	19.23%	< 0.001
Family Planning	8.18%	5.13%	0.214
Biological Concerns	13.64%	5.35%	0.002
Parent child communication about SRH issues	8.18%	5.56%	0.299
Sexually transmitted infections	26.36%	3.85%	< 0.001
Condoms	19.09%	3.63%	< 0.001
General information about sexual activity	23.64%	2.99%	< 0.001
Romantic relationships between partners	10.91%	2.35%	< 0.001
Any concern	63.64%	31.84%	< 0.001

## Discussion

The results of this study provide several insights into the SRH needs of unmarried adolescents in Jordan and the services they receive, and offer evidence that could be used to improve SRH services for youth in Jordan. Most importantly, our results suggest that a substantial proportion of HCPs have provided SRH services to unmarried youth, with more than 60% of practitioners reporting having seen at least one unmarried adolescent client for SRH-related concerns, which is in contrast to previous research and the common perception that unmarried youth in Jordan do not seek SRH services as a result of the conservative socio-cultural environment [2]. This is particularly important in an environment where information from parents and schools is extremely limited, as several studies have shown in Jordan [2, 8]. Furthermore, our findings deepen the understanding of HCP experiences and demand for services among youth, and highlight the urgency of supporting practitioners with adequate resources to deliver high quality, unbiased, youth-friendly SRH care in order to attract and retain young clients [10, 11].

Puberty was the most common topic reported by providers in our study, which would be expected, given the age range of the youth in question. Our results also suggest that family planning is an important concern among unmarried adolescent girls in Jordan, which at first may be somewhat surprising given the conservative setting; however, past research has found that young women in Jordan define family planning as a broader concept than just contraceptive use, as is often the focus of many programmatic initiatives in this domain, to include a wide range of topics related to the timing and spacing of pregnancies [6]. The strong social expectations surrounding marriage and childbearing in Jordan, combined with the fact that that child marriage is still common among certain groups of young women, may explain why planning for their future families is a topic of importance to young, unmarried women.

Further, our results show that a relatively small percentage of HCPs reported seeing unmarried youth with regard to information or services related to sexual activity, condom use and STIs, which is again in line with expectations given the context. While the percentage was small, the needs exist, thus emphasizing the urgency for more research with both youth and providers to better understand the challenges the unmarried youth population in Jordan face.

Our results also emphasize the importance of a clear and supportive policy environment to ensure that HCPs are aware of youth SRH service delivery guidelines, as well as ensuring that HCPs are adequately trained to provide SRH services to youth. We found that HCPs who were aware of service delivery guidelines related to youth SRH were significantly more likely to report having seen a youth client of either sex for such services than those who were unaware of any guidelines, while HCPs who received training on reproductive health were more likely to have reported seeing an unmarried female youth client than those who had not received any training. One potential explanation for these results is that HCPs who are aware of the guidelines or receive specific training may be more open to seeing unmarried clients, or may be more confident in their ability to provide them with care. In Jordan, pre-service training of HCPs on SRH issues, especially family planning counselling and on administering SRH services to youth, is very limited and has been previously documented as an important gap [2]. Given the conservative social environment, some HCPs may be disincentivized to provide unmarried youth with SRH services due to fear of professional repercussions or as a result of intermixing personal values with their professional duties, despite there being no explicit policy in Jordan that prohibits SRH service provision to unmarried youth. Furthermore, HCPs lacking clear knowledge as to SRH guidelines or national policy relating to youth SRH service delivery, may turn an unmarried client away, require parental or spousal permission unnecessarily, or enforce non-existent or unclear policies or laws based on age, parity, or marital status [12–16].

Last, several findings emerge from our study that relate to the alignment (or lack thereof) of provider and client sex, which may offer important and context specific insights related to youth-friendly SRH services. In our results, provider-client match with regard to sex appeared to be most important for female clients seeking services for puberty-related concerns, while conversely, female clients were more likely to see a male provider for services related to STIs and sexual activity. These results contrast somewhat with those of other studies in the literature that showed that young women tend to prefer female HCPs for SRH services [17–20]. We have identified several possible reasons why these patterns may have emerged in our data. The vast majority of midwives and nurses in our study sample were women, while the majority of physicians were men. As such, our results may simply be an artifact of representation by sex in certain cadres of HCPs, if female youth felt it was more appropriate to go to a physician, instead of nurses or midwives, for certain services based on perceptions of provider skills, expertise, or scopes of practice. In Jordan, nurses and midwives are not allowed to prescribe contraceptives or treat STIs*.* It is also possible that female HCPs could be perceived as being more deeply entrenched in the social norms that associate adolescent sexuality with shame, which may cause female youth to feel less comfortable seeking care from them regarding subjects that are considered taboo for unmarried youth. Other studies have found that nurses in particular may feel tension between their professional and personal identities as parents and community members, and may have difficulty separating the personal aspects of their life from their practice [16, 21]. Given the importance of honor in the Jordanian context, which is often tied to female sexual conduct, young women may avoid seeking services from HCPs that are perceived to be more embedded within the community, hence the preference for male HCPs for services that relate more directly to sexual activity. Future studies should seek to disentangle preferences for provider sex and type among youth for different types of services, which could facilitate improved service delivery.

With regard to male youth, our results suggest that fewer HCPs have experience providing services to young men. Previous studies in Jordan have reported that male youth believe that the type of health center where our study was conducted are only for women. Our results seem to support those findings, given that relatively few HCPs reported ever seeing a male youth client [2]. Other studies have also highlighted that many young men in Jordan think that SRH is only a concern for women [22]. Taken together, these results highlight the importance of shifting gender norms to encourage young men to become more actively engaged in matters related to SRH, while simultaneously ensuring that the service delivery environment is sensitive to the existing gendered context, that it is welcoming to young men, and that programs specifically target young men to improve their knowledge as to where they can seek SRH services.

This study has several strengths and limitations that we would like to highlight. Our sample of HCPs is large and reflects a wide range of professional cadres and provider characteristics. This study though adds a different dimension to the understanding of youth SRH in a conservative context by asking providers about their experiences rather than asking youth directly about their needs, perceptions or behaviors. In this context, practitioners may be more willing to report seeing unmarried youth for SRH services than youth themselves, as unmarried young people might be more affected by social desirability bias, fearing shame or punishment should they admit to seeking such services, even in the context of a study that ensures confidentiality. Furthermore, providers may feel more comfortable answering questions about their experience providing services to unmarried youth on an anonymous paper-based survey than through other means. That said, while we emphasized confidentiality during the consent process and all surveys were self-administered, some HCPs may not report seeing youth clients for fear of professional repercussions, even though providing SRH services to unmarried youth is allowed in the Jordanian context. In terms of limitations, while the majority of HCPs reported they had seen an unmarried youth client for SRH concerns, we cannot tell from the data the frequency with which youth come in with these concerns or whether they were accompanied by a parent/guardian. Additionally, our results should not be interpreted as service volume or as providing data on the volume of youth seeking SRH services, given that providers were only asked if they had seen at least one unmarried client. Furthermore, our survey did not ask providers details of their encounter with these unmarried youth, including if they delivered services, such as counseling or other support, for these SRH concerns, as our study is primarily interested in whether providers had encounters with youth related to these topical areas. Future research could examine more specifically the content of counseling and clinical services provided. Our results also are not able to differentiate if HCP’s saw more female or male clients because there was a higher demand from this group or because the providers were more willing to provide services to this group. Finally, while our study includes a large sample of HCPs, our results may not be generalizable to all of Jordan, as we focused in four communities in the Middle and Northern region of the country. In addition, our survey did not include pharmacies or private sector service providers, which is an important source of healthcare in Jordan [23].

## Conclusion

Unmarried youth in Jordan represent an important and understudied population with a range of SRH needs, especially in relation to puberty, family planning, and use of condoms. Ensuring that both the policy and service delivery environment supports the provision of youth-friendly SRH services is essential to meeting the SRH needs of Jordan’s youth; to that end, more research is needed to understand provider-client dynamics as well as the specific barriers that both youth and practitioners face in terms of service utilization and provision in this conservative environment.

## Data Availability

The datasets analyzed during the current study are not publicly available due to human subjects’ protections, but are available from the corresponding author on reasonable request.
